# Social interaction can select for reduced ability

**DOI:** 10.1098/rspb.2022.1788

**Published:** 2022-10-26

**Authors:** John M. McNamara, Max Wolf

**Affiliations:** ^1^ School of Mathematics, University of Bristol, Fry Building, Woodland Road, Bristol BS8 1UG, UK; ^2^ Department of Biology and Ecology of Fishes, Leibniz Institute of Freshwater Ecology and Inland Fisheries, Müggelseedamm 310, Berlin 12587, Germany; ^3^ Science of Intelligence, Research Cluster of Excellence, Marchstr. 23, Berlin 10587, Germany

**Keywords:** public goods game, polymorphism, evolutionary game theory, personality, comparative advantage

## Abstract

Animals, including humans, differ in a wide range of physical and cognitive abilities ranging from measures of running speed and physical strength to learning ability and intelligence. We consider the evolution of ability when individuals interact pairwise over their contribution to a common good. In this interaction, the contribution of each is assumed to be the best given their own ability and the contribution of their partner. Since there is a tendency for individuals to partially compensate for a low contribution by their partner, low-ability individuals can do well. As a consequence, for benefit and cost structures for which individuals have a strong response to partner’s contribution, there can be selection for reduced ability. Furthermore, there can be disruptive selection on ability, leading to a bimodal distribution of ability under some modes of inheritance.

## 1. Introduction

Humans differ in a wide range of physical and cognitive abilities ranging from measures of running speed and physical strength to learning ability and intelligence [1–6]; similar differences can be found in many other animal species [7–12]. One might naively expect the fitness benefit (payoff) that an individual receives when performing a specific task would increase with its ability at the task. While this will be true if the task is a ‘game against nature’, in this paper, we show that this need not always be the case when the task involves a competitive interaction with others.

Many decisions are made in a setting in which the best action of an individual depends on the actions of others. As an example, suppose that two parents provision their common young. Then assuming diminishing returns for increased provisioning (at high levels of provisioning) and individual costs of provisioning, the optimal rate of provisioning by one parent increases as the rate of provisioning of its partner decreases. If individuals respond to the provisioning rate of their partner by changing their own provisioning rate, then a low-ability parent may do well since its partner may partially compensate for its lack of provisioning. In fact, as we show, under some assumptions about responding, low-ability individuals may do better than high-ability individuals in the population.

We present a model in which the ability of an individual is genetically determined. Individuals from a large population meet pairwise at random and play a public goods game. Each adjusts its contribution to the common good to be the best given its own ability and the contribution of its partner, so that contributions are in Nash equilibrium. We investigate the evolution of ability under this assumption. As we will show, for some cost structures, selection can lead to reduced ability in the population. Furthermore, there can be disruptive selection so there is a tendency for the evolved distribution of abilities to be bimodal.

As with all modelling, conclusions depend critically on assumptions. We return to examine these assumptions in the Discussion. Our main effects rely on individuals revealing their true ability to their partner. This might be because ability is directly observable (as when ability increases with body size). Alternatively, ability might be revealed through behaviour. As we discuss, there is then selection on individuals to hide their ability if they are able to do so—a possible focus for future work.

## 2. The model

We consider a large population where each individual is characterized by an ability trait *a*, where 0 ≤ *a* ≤ 1. Individuals meet pairwise at random (they cannot choose their partner) and play a public goods game in which each chooses the effort they expend on the common good. The payoff to an individual of ability $\tilde{a}$ that expends effort $\tilde{e}$ when partner expends effort *e* is

2.1 $$W_{\tilde{a}}(\tilde{e},e)=B(\tilde{e}+e)-K(\tilde{a},\tilde{e}),$$

where the common benefit $B(\tilde{e}+e)$ depends on the sum of the efforts of both individuals and the cost paid $K(\tilde{a},\tilde{e})$ depends on an individual’s ability and effort. We assume that the common benefit is an increasing but decelerating function of the total effort. The cost paid is an increasing and accelerating function of the effort, with the cost paid for a given level of effort decreasing with ability. The marginal cost of extra effort is less for a high-ability individual than for a low-ability individual (electronic supplementary material, section S1.1). In economic terms, high-ability individuals have a comparative advantage [13]. Figures are based on the functions

2.2 $$\begin{array}{r} B(e_{\mathrm{tot}})=2e_{\mathrm{tot}}-{\left(e_{\mathrm{tot}}\right)}^{2}\;\mathrm{for}\;e_{\mathrm{tot}}<1,\mathrm{with}\;B(e_{\mathrm{tot}})=1\;\mathrm{for}\;e_{\mathrm{tot}}\geq 1, \end{array}$$

and

2.3 $$K(a,e)=(2-a)\;e^{p},$$

where the cost parameter *p* satisfies *p* > 1. These functions are illustrated in electronic supplementary material, section S1.2.

We assume that when individuals of abilities $\tilde{a}$ and *a* pair, the effort of each maximizes its payoff given its own ability and the effort of its partner. This characterization uniquely specifies the Nash equilibrium efforts $e^{*}(\tilde{a},a)$ and $e^{*}(a,\tilde{a})$ of the $\tilde{a}$ and *a* individuals, respectively (electronic supplementary material, section S3).

Ability is genetically determined, and is hence inherited (with possible mutation). We consider the evolution of ability using the framework of adaptive dynamics [14]. Suppose that almost all population members have ability *a*; i.e. *a* is the resident population strategy. A rare mutant with ability $\tilde{a}$ almost always pairs with a resident, so that the payoff to the mutant is

2.4 $$V(\tilde{a},a)=B(e^{*}(\tilde{a},a)+e^{*}(a,\tilde{a}))-K(\tilde{a},e^{*}(\tilde{a},a)).$$

## 3. Invasion fitness results

**Figure 1.**
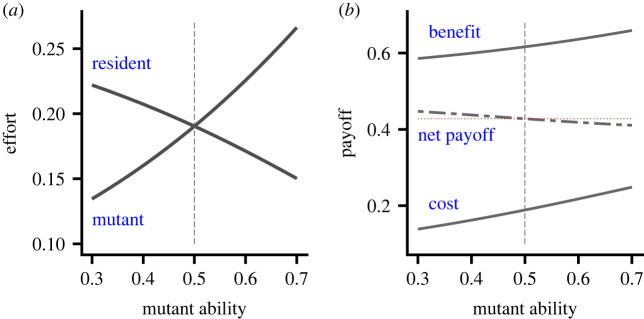
The effect of the ability of a single rare mutant individual on behaviour and performance. Resident population members all have ability *a* = 0.5. (*a*) The Nash equilibrium efforts of the mutant and its resident partner. (*b*) The benefit, cost and net payoff of the mutant. The payoff to a resident (horizontal dotted red line) is also shown. Benefit and cost functions given by equations (2.2) and (2.3) where *p* = 1.25. (Online version in colour.)

Here, we consider a single mutant individual within a resident population. We analyse how the effort and payoff of the mutant changes as its ability increases. In general, the effort of a mutant increases and the effort of its resident partner decreases as the ability of the mutant increases (electronic supplementary material, section S3.4). The sum of the efforts of the pair, and hence value of the common good, increases as mutant ability increases (electronic supplementary material, section S3.4). Figure 1 illustrates a specific case. In this case, the changes in efforts are so strong that the mutant’s payoff decreases with its ability.

**Figure 2.**
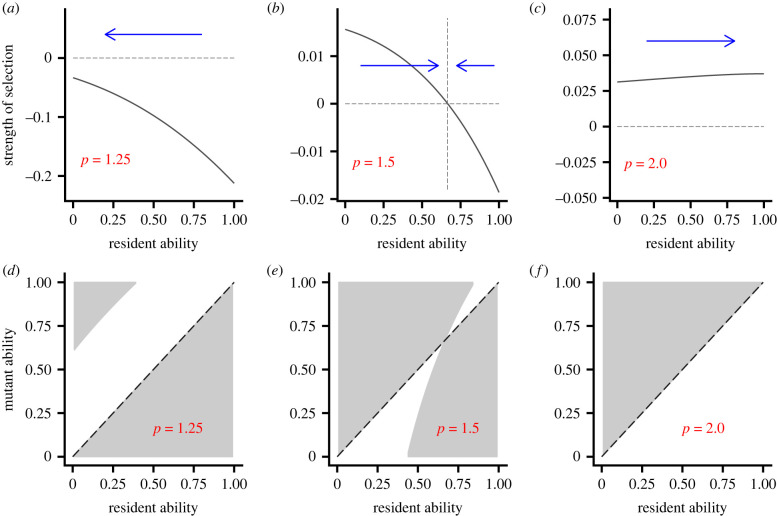
Invasion analysis for three cost functions. *K* is given by equation (2.3) with *p* = 1.25 in (*a*,*d*), *p* = 1.5 in (*b*,*e*) and *p* = 2.0 in (*c*,*f*). (*a*–*c*) The strength and direction of selection on ability (electronic supplementary material, section S4.1). (*d*–*f*) Pairwise invasibility plots [14]. In these plots, an ability is the resident ability if almost all population members have this ability. The grey area indicates when a rare mutant can invade the resident population. Benefit function given by equation (2.2). (Online version in colour.)

Figure 2*a* shows the strength of selection on ability (electronic supplementary material, section S4.1) for cost parameter *p* = 1.25. In this case, selection is in the direction of reduced ability for all abilities, so that *a* = 0 is the unique convergence stable ability. The full pairwise invasibility plot (PIP [14]) shows that for high resident ability all lower-ability mutants do better than residents, while some high-ability mutants can invade when the resident ability is low (figure 2*d*). Figure 2*b*,*e* shows the corresponding plots for the case *p* = 1.5. In this case, there is selection to a convergence stable value of ability that lies between 0 and 1, with disruptive selection at this convergence stable point. For higher values of *p* there can be selection for maximization of ability, so that *a* = 1 is the unique convergence stable point (figure 2*c*,*f*).

The above cases illustrate that selection may or may not be for reduced ability, depending on the cost-benefit structure. If individuals were playing a ‘game against nature’, in that their abilities and actions did not affect the action of their partner, then selection would always be towards increased ability (electronic supplementary material, section S2). Here, however, we are considering a game in which the partner adjusts its effort in response to the effort of the focal individual. The degree of adjustment is then crucial (electronic supplementary material, section S4.2). When the cost function is given by equation (2.3) responsiveness is greater in the case *p* = 1.25 than when *p* = 2.0 (electronic supplementary material, section S4.3), leading to the difference in results in these cases.

## 4. Evolutionary simulation results

**Figure 3.**
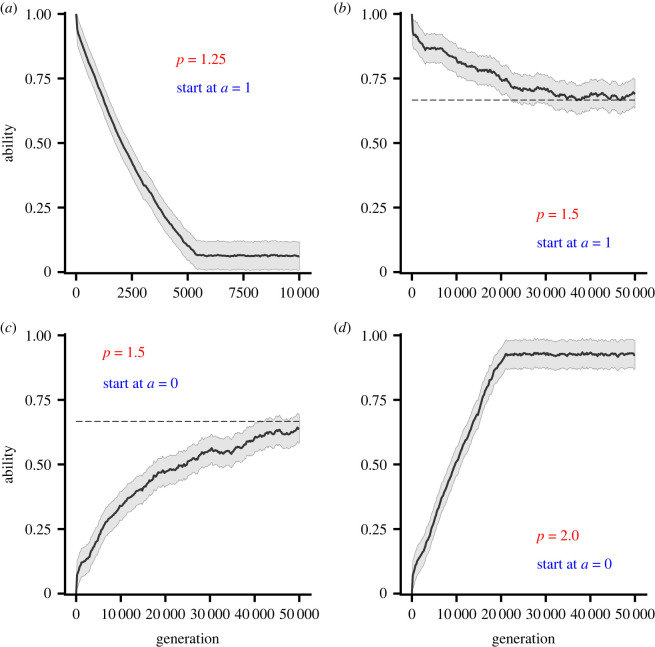
Evolution of ability simulated for a sexually reproducing population. Plots show the median ability and 95% quantiles. The cost *K* is given by equation (2.3) with *p* = 1.25 in (*a*), *p* = 1.5 in (*b*,*c*) and *p* = 2.0 in (*d*). In (*a*) and (*b*) initially all population members have ability *a* = 1. In (*c*,*d*) initially all population members have ability *a* = 0. Benefit function given by equation (2.2). (Online version in colour.)

**Figure 4.**
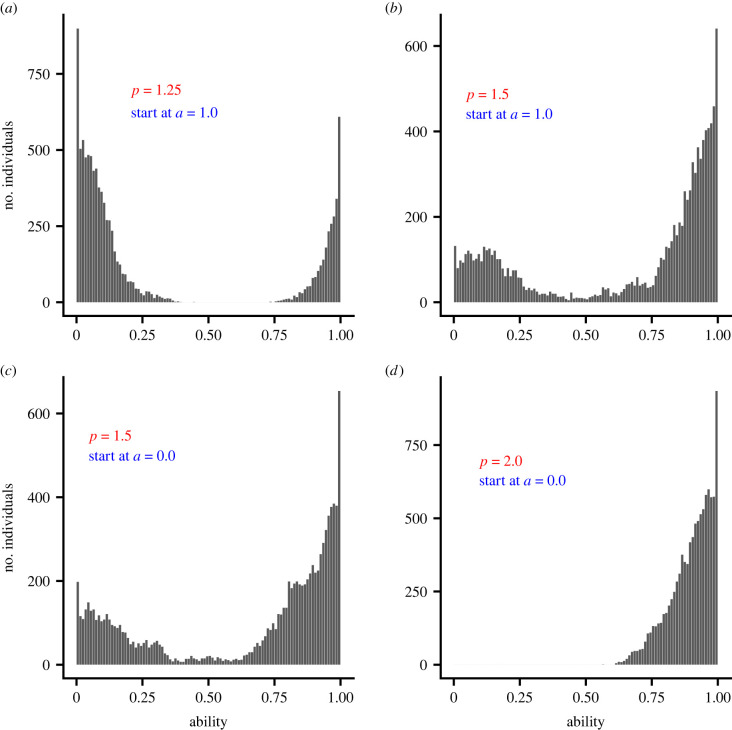
Evolution of ability simulated for an asexually reproducing population. Plots show the distribution of abilities after 10 001 generations. The cost *K* is given by equation (2.3) with *p* = 1.25 in (*a*), *p* = 1.5 in (*b*) and (*c*), and *p* = 2.0 in (*d*). In (*a*,*b*) initially all population members have ability *a* = 1. In (*c*,*d*) initially all population members have ability *a* = 0. Benefit function given by equation (2.2). (Online version in colour.)

We complement our invasion analysis with evolutionary simulations in which ability is allowed to evolve (electronic supplementary material, section S5). When reproduction is sexual, in each of the cases *p* = 1.25, 1.5 and 2.0 median ability evolves to (approximately) the corresponding convergence stable value, regardless of initial ability (figure 3). In these cases, the form of inheritance (the infinitesimal model [15]—see electronic supplementary material, section S5) ensures a unimodal distribution of abilities at all times. In contrast, if reproduction is asexual, a bimodal distribution of abilities evolves when the corresponding PIP plots indicate disruptive selection (figure 4).

## 5. Discussion

We have analysed a model in which population members pair up to play a public goods game. Within a pair, the contribution of each individual to the common good (its effort) is assumed to be the best given its own ability and the effort of its partner (efforts are in Nash equilibrium). We have assumed that the common benefit function has diminishing returns, so that it is optimal to compensate for a partner’s lack of effort by increasing own effort. When pair members are highly responsive to each others’ efforts, with a strong tendency to compensate for the lack of effort of the partner, we have shown there can be selection to reduce ability. Our results are in line with recent research finding that neglecting key state variables underlying behaviour (here: ability) can give rise to dramatically different predictions [16–18].

Our assumption that the effort expended on the common benefit has diminishing returns seems reasonable in many situations. For example, when two parents care for their common young, we might equate effort with the rate of food provisioning to the young. Survival of the young would then typically increase with the sum of the efforts of the parents, but there would be diminishing returns. In this example, we could equate ability with foraging ability, and might expect a parent to partially compensate for the poor provisioning rate of a low-ability partner by increasing its own effort (cf. [19]). Although we have restricted our analysis to two-player public goods games, there are many other situations in which low ability might be advantageous. For example, if a group needs to remain vigilant for predators while foraging, and one individual is known to be poor at spotting predators because of poor eyesight, then it might be worth for others to increase their vigilance to compensate. The poor eyesight individual may then spend little time on vigilance and get more food than other group members. Similarly, if a group is defending its territory against intruders, then fighting ability might equate with size. The Nash equilibrium strategy of group members might then be to have the largest individuals at the fore of the fighting. Small, low-ability individuals may then do better than larger group members. These examples suggest that there could be selection for reduced ability in other settings.

Our model ignores effects of ability on fitness outside the focal public goods game. These residual fitness effects will tend to counteract any advantage to low ability in the public good game. Evolved levels of ability will be determined by both factors, as well as deleterious mutational effects that will tend to reduce ability. Phenotypic ability may also be affected by developmental noise [20].

In those cases investigated, when there is selection for reduced ability there is also disruptive selection. The distribution of abilities that is predicted to evolve then depends strongly on the genetic system and mode of inheritance [21]. We have illustrated two extreme cases (figures 3 and 4), but whatever the form of inheritance, we might expect disruptive selection will tend to broaden the distribution of abilities.

We have assumed that population members pair at random. If instead we had allowed partner choice, then predictions would depend on the distribution of abilities in the population. If we consider evolution in an essentially monomorphic population, then there is little variation in ability and hence little to be gained by being choosy. For this reason, the equation for adaptive dynamics (that describes the evolution of ability in an essentially monomorphic population) is unaltered if there are any costs of rejecting a partner, although pairwise invasibility plots are altered (electronic supplementary material, section S4.4). However, if there were substantial variability in ability in a population, as in our simulation of evolution in an asexual population (figure 4), then allowing partner choice might have a strong effect, especially if the costs of being choosy were small. This could be investigated using a sequential choice model in which acceptance thresholds and ability co-evolve.

The assumption that efforts are in Nash equilibrium is key to our predictions. This assumption may be reasonable when individuals know the ability of their partner (as well as their own ability). Rather than assuming a Nash equilibrium, we have additionally investigated the co-evolution of ability and the rules for choosing effort in a pair when abilities are known (electronic supplementary material, section S6). This model again predicts evolution to low abilities when the cost parameter is *p* = 1.25. In some cases, such as fighting ability, the size of partner may be highly correlated with ability, so that the assumption that individuals know the ability of their partner seems reasonable. This may explain how and why differences in many physical abilities like physical strength and fighting ability—which can be assessed visually by humans and other animals [22–25]—can emerge and be maintained by natural selection, despite their apparent fitness disadvantages.

Differences in cognitive abilities are widespread in the animal kingdom, ranging from individual differences in particular cognitive abilities to differences in general cognitive abilities, and such differences are heritable and correlated to fitness [26–29]. Cognitive abilities may be recognized by interaction partners (especially in repeated interactions—but see below) and whenever this is the case, the same mechanism that we described above could trigger reduced ability or the emergence of adaptive differences in cognitive abilities between individuals.

Some abilities, such as aspects of cognitive ability, may not be easily observed, but are potentially revealed by the performance of an individual in a task. It may then be advantageous for individuals of high ability to hide their ability by deliberately performing poorly in the task, even though this incurs an immediate cost. Previous models have predicted this behaviour during parental care [30], during reputation formation [31] and in a public goods game [32]. Further work might investigate whether individuals are predicted to deliberately perform poorly in one task (which may not be a game) in order to gain an advantage when they interact socially over another task.

## Data Availability

The programmes used to generate the figures can be found at Zenodo (doi:10.5281/zenodo.7143007) [33]. The data are provided in electronic supplementary material [34].

## References

[1] Archer J, Thanzami V. 2007 The relation between physical aggression, size and strength, among a sample of young Indian men. Pers. Individ. Dif. **43**, 627-633. (10.1016/j.paid.2007.01.005)

[2] Carroll JB, Maxwell SE. 1979 Individual differences in cognitive abilities. Annu. Rev. Psychol. **30**, 603-640. (10.1146/annurev.ps.30.020179.003131) 20738189

[3] Herrmann E, Hernández-Lloreda MV, Call J, Hare B, Tomasello M. 2010 The structure of individual differences in the cognitive abilities of children and chimpanzees. Psychol. Sci. **21**, 102-110. (10.1177/0956797609356511) 20424030

[4] Kanai R, Rees G. 2011 The structural basis of inter-individual differences in human behaviour and cognition. Nat. Rev. Neurosci. **12**, 231-242. (10.1038/nrn3000) 21407245

[5] Plomin R, Spinath FM. 2002 Genetics and general cognitive ability (g). Trends Cogn. Sci. **6**, 169-176. (10.1016/S1364-6613(00)01853-2) 11912040

[6] Watkins CD, Fraccaro PJ, Smith FG, Vukovic J, Feinberg DR, Debruine LM, Jones BC. 2010 Taller men are less sensitive to cues of dominance in other men. Behav. Ecol. **21**, 943-947. (10.1093/beheco/arq091)

[7] Bolnick DI, Svanbäck R, Fordyce JA, Yang LH, Davis JM, Hulsey CD, Forister ML. 2003 The ecology of individuals: incidence and implications of individual specialization. Am. Nat. **161**, 1-28. (10.1086/343878) 12650459

[8] Banerjee K, Chabris CF, Johnson VE, Lee JJ, Tsao F, Hauser MD. 2009 General intelligence in another primate: individual differences across cognitive task performance in a New World monkey (*Saguinus oedipus*). PLoS ONE **4**, e5883. (10.1371/journal.pone.0005883) 19536274 PMC2690653

[9] Ehlinger TJ, Wilson DS. 1988 Complex foraging polymorphism in bluegill sunfish. Proc. Natl Acad. Sci. USA **85**, 1878-1882. (10.1073/pnas.85.6.1878) 16578831 PMC279884

[10] Galsworthy MJ, Paya-Cano JL, Monleón S, Plomin R. 2002 Evidence for general cognitive ability (g) in heterogeneous stock mice and an analysis of potential confounds. Genes Brain Behav. **1**, 88-95. (10.1034/j.1601-183X.2002.10204.x) 12884979

[11] Griffin AS, Guillette LM, Healy SD. 2015 Cognition and personality: an analysis of an emerging field. Trends Ecol. Evol. **30**, 207-214. (10.1016/j.tree.2015.01.012) 25736691

[12] Sih A, Sinn DL, Patricelli GL. 2019 On the importance of individual differences in behavioural skill. Anim. Behav. **155**, 307-317. (10.1016/j.anbehav.2019.06.017)

[13] Ricardo D. 1817 On the principles of political economy and taxation. London, UK: J. Murray.

[14] Geritz SAH, Kisdi E, Meszena G, Metz JAJ. 1998 Evolutionarily singular strategies and the adaptive growth and branching of evolutionary trees. Evol. Ecol. **12**, 35-37. (10.1023/A:1006554906681)

[15] Barton NH, Etheridge AM, Véber A. 2017 The infinitesimal model: definition, derivation, and implications. Theor. Popul. Biol. **118**, 50-73. (10.1016/j.tpb.2017.06.001) 28709925

[16] McNamara JM. 2013 Towards a richer evolutionary game theory. J. R. Soc. Interface **10**, 20130544. (10.1098/rsif.2013.0544) 23966616 PMC3785819

[17] McNamara JM, Wolf M. 2015 Sexual conflict over parental care promotes the evolution of sex differences in care and the ability to care. Proc. R. Soc. B **282**, 20142752. (10.1098/rspb.2014.2752) PMC4345446 25694618

[18] Wolf M, McNamara JM. 2012 On the evolution of personalities via frequency-dependent selection. Am. Nat. **179**, 679-692. (10.1086/665656) 22617258

[19] Harrison F, Barta Z, Cuthill I, Székely T. 2009 How is sexual conflict over parental care resolved? A meta-analysis. J. Evol. Biol. **22**, 1800-1812. (10.1111/j.1420-9101.2009.01792.x) 19583699

[20] Wilson DS. 1998 Adaptive individual differences within single populations. Phil. Trans. R. Soc. Lond. B **353**, 199-205. (10.1098/rstb.1998.0202)

[21] Rueffler C, Van Dooren TJ, Leimar O, Abrams PA. 2006 Disruptive selection and then what? Trends Ecol. Evol. **21**, 238-245. (10.1016/j.tree.2006.03.003) 16697909

[22] Arnott G, Elwood RW. 2009 Assessment of fighting ability in animal contests. Anim. Behav. **77**, 991-1004. (10.1016/j.anbehav.2009.02.010)

[23] Huntingford FA, Turner A 1987 Animal conflict. London, UK: Chapman & Hall.

[24] Sell A, Cosmides L, Tooby J, Sznycer D, von Rueden C, Gurven M. 2009 Human adaptations for the visual assessment of strength and fighting ability from the body and face. Proc. R. Soc. B **276**, 575-584. (10.1098/rspb.2008.1177) PMC2664345 18945661

[25] Sell A, Bryant GA, Cosmides L, Tooby J, Sznycer D, von Rueden C, Krauss A, Gurven M, 2010 Adaptations in humans for assessing physical strength from the voice. Proc. R. Soc. B **277**, 3509-3518. (10.1098/rspb.2010.0769) PMC2982226 20554544

[26] Deary IJ, Penke L, Johnson W. 2010 The neuroscience of human intelligence differences. Nat. Rev. Neurosci. **11**, 201-211. (10.1038/nrn2793) 20145623

[27] Knopik VS, Neiderhiser JM, DeFries JC, Plomin R. 2017 Behavioral genetics. New York, NY: Worth Publishers.

[28] Boogert NJ, Fawcett TW, Lefebvre L. 2011 Mate choice for cognitive traits: a review of the evidence in nonhuman vertebrates. Behav. Ecol. **22**, 447-459. (10.1093/beheco/arq173)

[29] Boogert NJ, Madden JR, Morand-Ferron J, Thornton A. 2018 Measuring and understanding individual differences in cognition. Phil. Trans. R. Soc. B **373**, 20170280. (10.1098/rstb.2017.0280) 30104425 PMC6107562

[30] McNamara JM, Gasson CE, Houston AI. 1999 Incorporating rules for responding into evolutionary games. Nature **401**, 368-371. (10.1038/43869) 10517633

[31] McNamara JM, Doodson P. 2015 Reputation can enhance or suppress cooperation through positive feedback. Nat. Commun. **6**, 6134. (10.1038/ncomms7134) 25601004

[32] Leimar O, McNamara JM. 2019 Learning leads to bounded rationality and the evolution of cognitive bias in public goods games. Sci. Rep. **9**, 16319. (10.1038/s41598-019-52781-7) 31705040 PMC6841956

[33] McNamara J. 2022 Software for 10.1098/rspb.2022.1788. Zenodo. (10.1098/rspb.2022.1788)

[34] McNamara JM, Wolf M. 2022 Social interaction can select for reduced ability. Figshare. (10.6084/m9.figshare.c.6238479) PMC9579777 36259207

